# Abnormal Intrinsic Functional Hubs in Severe Male Obstructive Sleep Apnea: Evidence from a Voxel-Wise Degree Centrality Analysis

**DOI:** 10.1371/journal.pone.0164031

**Published:** 2016-10-10

**Authors:** Haijun Li, Lan Li, Yi Shao, Honghan Gong, Wei Zhang, Xianjun Zeng, Chenglong Ye, Si Nie, Liting Chen, Dechang Peng

**Affiliations:** 1 Department of Radiology, the First Affiliated Hospital of Nanchang University, Nanchang 330006, Jiangxi Province, China; 2 School of Public Health, Nanchang University, Nanchang 330006, Jiangxi Province, China; 3 Department of Ophthalmology, the First Affiliated Hospital of Nanchang University, Nanchang 330006, Jiangxi Province, China; 4 Department of Pneumology, the First Affiliated Hospital of Nanchang University, Nanchang 330006, Jiangxi Province, China; University of Texas at Austin, UNITED STATES

## Abstract

**Purpose:**

Obstructive sleep apnea (OSA) has been associated with changes in brain structure and regional function in certain brain areas. However, the functional features of network organization in the whole brain remain largely uncertain. The purpose of this study was to identify the OSA-related spatial centrality distribution of the whole brain functional network and to investigate the potential altered intrinsic functional hubs.

**Methods:**

Forty male patients with newly confirmed severe OSA on polysomnography, and well-matched good sleepers, participated in this study. All participants underwent a resting-state functional MRI scan and clinical and cognitive evaluation. Voxel-wise degree centrality (DC) was measured across the whole brain, and group difference in DC was compared. The relationship between the abnormal DC value and clinical variables was assessed using a linear correlation analysis.

**Results:**

Remarkably similar spatial distributions of the functional hubs (high DC) were found in both groups. However, OSA patients exhibited a pattern of significantly reduced regional DC in the left middle occipital gyrus, posterior cingulate cortex, left superior frontal gyrus, and bilateral inferior parietal lobule, and DC was increased in the right orbital frontal cortex, bilateral cerebellum posterior lobes, and bilateral lentiform nucleus, including the putamen, extending to the hippocampus, and the inferior temporal gyrus, which overlapped with the functional hubs. Furthermore, a linear correlation analysis revealed that the DC value in the posterior cingulate cortex and left superior frontal gyrus were positively correlated with Montreal cognitive assessment scores, The DC value in the left middle occipital gyrus and bilateral inferior parietal lobule were negatively correlated with apnea-hypopnea index and arousal index in OSA patients.

**Conclusion:**

Our findings suggest that OSA patients exhibited specific abnormal intrinsic functional hubs including relatively reduced and increased DC. This expands our understanding of the functional characteristics of OSA, which may provide new insights into understanding the dysfunction and pathophysiology of OSA patients.

## Introduction

Obstructive sleep apnea (OSA) is a common respiratory disorder characterized by repeated collapses of the upper airway, causing episodes of airflow cessation (apnea), or decreases in airflow (hypopnea) during sleep that are associated with intermittent hypoxemia, hypercapnia, and fragmented sleep[1]. A review of epidemiological studies quotes the mean prevalence of OSA as 4% (range 1–17%) in women and 6% (range 3–18%) in men, and this increases with age and over time according to the published literature from 1993 to 2013[2]. OSA has been associated with a broad range of detrimental effects including excessive daytime sleepiness, increased risk of industrial and vehicular accidents, impaired work performance, increased incidence of diabetes, hypertension, congestive heart failure, stroke, and cardiovascular mortality, and a reduced quality of life[1,3,4]. An increasing number of studies have found that OSA was associated with cognitive dysfunction, particularly in memory, attention, learning, and executive function[5–7]. Comprehensive knowledge of the central nervous system in OSA patients is important to understand the evolution of cognitive dysfunction and provide novel interventional treatments that are likely to benefit these patients. The current, prevailing view, is that sleep fragmentation and intermittent hypoxia are the underlying mechanisms of cognitive dysfunction in OSA patients[8–10]. However, the exact neurological basis of the development of neurocognitive dysfunction in patients with OSA is largely unknown. Neuroimaging methodologies can be used to explore the changes in the brain, and to improve our understanding of cognitive dysfunction in the patients with OSA.

Many previous structural neuroimaging studies, including voxel-based morphometry (VBM), and diffusion tensor imaging (DTI), have demonstrated that OSA patients may have brain tissue injury. This finding is mainly expressed as an abnormal regional gray matter volume or gray matter concentration, white matter integrity in multiple brain regions, including the hippocampus, cingulate cortex, limbic areas, basal ganglia, frontal lobe, temporal lobe, parietal lobe, and cerebellum, which are responsible for memory, autonomic, cognitive, and affective controls[11–16]. The structural changes are accompanied by impaired function. OSA patients showed abnormal local spontaneous activity using regional homogeneity[17] and amplitude of low frequency fluctuation[18], altered resting-state functional connectivity (FC) based on voxel-level independent component analysis[19], and disturbed regional connected neural networks using seed-based FC during the resting-state[20,21]. However, the human brain is intrinsically organized into multiple distinct yet inherently interacting functional networks[22,23]. Although brain structural damage and regional brain dysfunction in many brain regions was found in patients with OSA, as previously mentioned, they do not directly yield important functional network topological changes, for example, how the whole brain functionally interacts (i.e., connection weights among regions), and the complexity of the functional network of these interactions (i.e., brain network shape) during the resting-states.

In recent years, graph theoretical analysis has become an increasingly useful tool to characterize the complex system of functional brain networks, and provides a unique framework to test differences in the topological organization of brain networks[24], which is considered to be the physiological basis of information processing and mental representation[25].A graph theoretical analysis based on regional gray matter volume study found that OSA patients showed altered topological properties of brain structural networks, expressed as decreased local efficiency, and decreased regional properties in the left angular gyrus, the right lingual gyrus, and the inferior frontal gyrus[26]. In addition, OSA subjects showed complex, aberrant functional connectivities in the resting-state in various brain regions including the cerebellum, frontal, parietal, temporal, occipital, limbic, and basal ganglion regions that may underlie the impaired responses in autonomic, executive, cognitive, affective, and sensorimotor functions using inter-regional FC[27]. Using inter-regional large-scale brain connectivity analysis at the level of anatomical structures, these two studies have found that OSA patients have abnormal brain structure and function network, respectively. Each anatomical structural brain area contains many neurons, and has a different function. However, the functional network topological properties across the whole brain at the voxel level have not been directly revealed in OSA patients. Voxel-wise degree centrality (DC) is a graph theory-based network measurements tool at the voxel level, and DC represents the number of direct connections for a given voxel with the rest of the whole brain voxel rather than between specific nodes or regions. Voxel-wise DC can allow us to map the brain functional hubs (brain regions concentrated by large number of connections with the rest of whole brain) without a priori nodes or a region of interest[28]. Functional hubs play a pivotal role showing high cost and are especially vulnerable to aberrant disease conditions[29]. Compared with the previous resting-state fMRI studies that focus on regional functional measurements (i.e., the regional homogeneity[17], amplitude of low-frequency fluctuations[18], independent component analysis[19]), seed-based FC[20,21], and large-scale brain network analysis[27], the index of voxel-wise DC emphasizes the influence and importance of a given network at voxel level and reflects the functional brain network hub properties (i.e., the communication ability of network information). Previous research has confirmed that voxel-wise DC has a high sensitivity, specificity, and test-retest reliability[30], and it is increasingly used in psychiatric and neurodegenerative disorders[30–35].

In this study, our aim was to identify the spatial centrality distribution (hubs) of the whole brain functional network and to investigate the potential altered intrinsic functional hubs in male patients with severe OSA using voxel-wise DC. We hypothesized that OSA may exhibit impaired connectivity of the intrinsic functional hubs across the whole brain.

## Materials and Methods

### Subjects

The study was approved by the Medical Research Ethics Committee of The First Affiliated Hospital of Nanchang University. Written informed consent was obtained from each participant before they participated in the study. We recruited forty male patients with newly diagnosed severe OSA according to the American Academy of Sleep Medicine guidelines[36] and 40 male age- and education-matched good sleepers (GSs) from the Sleep Monitoring Room of the Respiratory Department at the First Affiliated Hospital of Nanchang University, between June 2013 and December 2015. The inclusion criteria for the OSA patients and GSs were as follows: (1) male sex; (2) older than 20 but less than 60 years of age; (3) an apnea-hypopnea index (AHI) equal to or greater than 30 for OSA; or an AHI less than 5 for GSs. The exclusion criteria for both patients with OSA and GSs were as follows: (1) other sleep disorders, such as primary insomnia; (2) diabetes, and chronic obstructive pulmonary disease and heart diseases; (3) neurodegenerative diseases, history of cerebrovascular disease, traumatic brain injuries; (4) psychosis disorder, epilepsy; (5) illicit drug or alcohol abuse; (6) a structural lesion based on conventional MRI scans; and (7) MRI contraindications, such as claustrophobia, or metallic implants in the body. Each participant underwent a detailed clinical interview, self-reported sleep questionnaire, overnight polysomnography and an MRI scan.

### Polysomnography and neuropsychological assessments

All of the participants were required to abstain from drinking coffee or alcoholic beverages for one day prior to participation in this study. Full nocturnal polysomnography monitoring was performed using the Respironics LE-Series Physiological Monitoring System (Alice 5 LE, FL, USA). Overnight polysomnography was recorded from 10pm until 6am the next morning. According to the American Academy of Sleep Medicine guidelines, the standard electroencephalogram derivations (EEG: from frontal, central, occipital regions: F4/M1, C4/M1, O2/M1; and back-up derivations: F3/M2, C3/M2, and O1/M2), chin electromyogram (EMG: located in the three chin electrodes and the middle of the right anterior tibialis), electro-oculogram (EOG: located in the cornea and retina), and electrocardiogram (ECG) were recorded. The oral and nasal airflow, snoring, chest and abdominal breathing, oxygen saturation (SaO_2_), and body position were also recorded, along with total sleep time, sleep latency, sleep efficiency, arousal, and respiratory events. According to the American Academy of Sleep Medicine manual, an obstructive apnea was defined as a reduction in airflow ≥ 90% lasting at least 10 seconds and associated with persistent respiratory effort; hypopnea was defined as a reduction in airflow ≥ 30% lasting at least 10 seconds and accompanied by a 4% or greater oxygen desaturation[36]. The AHI was calculated as the average of the total number of apnea and hypopnea events experienced per hour of sleep. The arousal index (AI) was computed as the mean number of EEG arousals per hour of sleep.

All of the OSA patients and GSs were evaluated with a self-reported sleep questionnaire for excessive daytime sleepiness using the Epworth sleepiness scale (ESS), which asks the subject to rate his or her probability of falling asleep on a scale of increasing probability from 0 to 3 for eight different situations[37]. A score equal to or greater than 10 demonstrates excessive daytime sleepiness in OSA patients. Global cognitive function was evaluated using the Montreal cognitive assessment (MoCA) including naming, executive function, calculation, attention, language, memory, abstraction, and orientation, with a total MoCA score lower than 26 indicating cognitive impairment[38]. If the length of subjects’ education was less than 12 years, one point was added to the total score, as an education deviation adjustment[38].

### MRI data acquisition

All of the participants underwent MRI scans using a 3.0 Tesla MRI system with 8-channel head coil (Siemens, Erlangen, Germany). All of the participants were asked to quietly lie on the scanning bed, to clear their minds as much as possible, to keep their eyes closed, and to stay awake during MRI scans. Firstly, conventional T1-weighted imaging (repetition time (TR) = 250 ms, echo time (TE) = 2.46, slices = 19, slice thickness = 5mm, gap = 1.5 mm, field of view (FOV) = 220 mm × 220 mm) and T2-weighted imaging (TR = 4000 ms, TE = 113 ms, slices = 19, slice thickness = 5 mm, gap = 1.5 mm, FOV = 220 mm × 220 mm) were collected. Then, the resting-state functional MRI images were collected using an echo planar imaging (EPI) sequence with the following parameters: TR = 2000 ms, TE = 30 ms, FOV = 230 mm × 230 mm, thickness = 4.0 mm, gap = 1.2 mm, matrix = 64 × 64, and flip angle = 90°. Each patient run consisted of 240 volumes in 8 minutes, and each brain volume comprised 30 axial slices. Finally, high-resolution three-dimensional T1-weighted images were obtained using a magnetization-prepared rapid gradient echo sequence (TR = 1900 ms, TE = 2.26 ms, FOV = 250 mm × 250 mm, resolution matrix = 256 × 256, flip angle = 9°, thickness = 1.0 mm, gap = 0.5 mm, slices = 176). Foam pads were used to reduce head movements, and earplugs were used to minimize scanner noise during the MRI scan.

### MRI data preprocessing

Before preprocessing, in order to rule out macro structural brain lesions which may affect brain microstructure or function, all of the conventional T1-weighted imaging and T2-weighted imaging had been reviewed by two senior radiologists in the department of radiology of the First Affiliated Hospital of Nanchang University. All of the high-resolution T1-weighted images were displayed and carefully checked, and functional images were checked by MRIcro software (www.MRIcro.com) to exclude potential low image quality by two authors. No participants were excluded because of brain lesions or low image quality. The resting-state functional and structural images were preprocessed using Data Processing & Analysis for Brain Imaging (DPABI) (http://rfmri.org/DPABI) and Statistical Parametric Mapping (SPM8) (http://www.fil.ion.ucl.ac.uk/spm), which was run on the MATLAB2010a (Mathworks, Natick, MA, USA) platform. The first 10 functional volumes of each participant were removed to allow for the participants’ adaptation to the scanning noise and the stability of the initial signal, and finishing of slice timing. Three-dimensional head motion correction was conducted for the remaining time points. None of the participants were removed according to the head motion criteria, which included a maximum spin (x, y, z) of less than 2.0° and a maximum cardinal direction displacement (x, y, z) of less than 2.0 mm. Four patients with OSA were excluded according to the standard of head motion, meaning that frame-wise displacement (FD) was more than 2.5 standard deviations based on the method of Van Dijk et al[39]. The mean FD was not significantly different between the rest of patients with OSA and GSs (Table 1). Anatomical and functional images were manually reoriented to the anterior commissure to achieve better registration, and structural images were co-registered to the functional images for each individual using a linear transformation. In sequence, the transformed structural images were segmented into gray matter, white matter, and cerebrospinal fluid using the new segmentation in SPM8[40]. The Diffeomorphic Anatomical Registration Through Exponentiated Lie Algebra tool was used to compute transformations from native space to Montreal Neurological Institute (MNI) space, and re-sampled to 3 mm × 3 mm × 3 mm voxels. Subsequently, the white matter signal, cerebrospinal fluid signal, global signal, and Friston 24-parameter[41] were regressed from the time series of all voxels via linear regression. Finally, a temporal filter (0.01–0.08 Hz) was performed to reduce the effect of low-frequency drift and high-frequency noise.

**Table 1:** Demographic and clinical data between the patients with OSA and GSs.

Characteristic	OSA patients (N = 36)		GSs (N = 40)		*t-*value	*P*-value
	Mean	SD	Mean	SD		
Age, years	39.0	8.1	38.8	11.2	0.09	0.930
Education, years	11.8	3.4	11.0	3.5	0.95	0.348
BMI, kg/m^2^	27.8	3.5	23.1	2.1	7.09	< 0.001
Sleep onset latency, min	10.3	4.6	18.1	5.7	-6.48	< 0.001
Total sleep time, min	391.7	80.5	398.3	20.2	-0.50	0.618
Sleep efficiency, %	79.3	9.4	91.3	3.2	-7.58	< 0.001
AHI, /hour	56.5	19.0	2.5	1.3	17.92	< 0.001
Stage 1, %	31.3	19.4	10.2	3.5	6.73	< 0.001
Stage 2, %	38.7	15.7	39.6	6.4	0.33	0.740
Stage 3+4, %	22.4	19.2	29.9	5.2	-2.40	0.019
REM, %	7.6	8.3	21.0	6.7	-7.78	< 0.001
SaO_2_ < 90%, %	25.1	21.0	0.2	0.2	7.49	< 0.001
nadir SaO_2_, %	66.3	12.8	89.2	3.9	10.75	< 0.001
AI, /hour	38.8	21.3	11.7	2.9	7.96	< 0.001
ESS	12.0	4.0	3.3	2.3	11.80	< 0.001
MoCA	25.3	2.6	28.3	1.2	-6.65	< 0.001
Mean FD, mm	0.054	0.021	0.050	0.024	0.96	0.339

**Abbreviations:** OSA, obstructive sleep apnea; GSs, good sleepers; N, number; SD, standard deviation; BMI, body mass index; AHI, apnea-hypopnea index; REM, rapid eye movement; SaO_2,_ oxygen saturation; AI, arousal index; SaO_2_ < 90%, percentage of total sleep time spent at oxygen saturation less than 90%; ESS, Epworth sleepiness scale; MoCA, Montreal cognitive assessment; FD, framewise displacement.

### Voxel-wise degree centrality analysis

The voxel-wise DC was generated for the resting-state fMRI time series using the Resting-State fMRI Data Analysis Toolkit V1.8 (REST V1.8) (http://www.restfmri.net) in the default gray matter mask provided by DPABI (in the MNI-152 standard space with 3 mm × 3 mm × 3mm voxel size, 67541 voxels). Each voxel acted as a node, and all pairs of voxel correlations as the edge. For each participant, the Pearson’s correlation coefficients were computed between any pairs of voxels in the default mask so that the whole brain functional connectivity matrix was constructed. According to the adjacency matrix of a graph, voxel-wise DC can be computed as in Eq (1) [28]

$$DC\left(i\right)=\sum_{j=1}^{N}r_{ij}\left(r_{ij}>r_{0}\right)$$ (1)

where r_ij_ is the correlation coefficient between voxel i and voxel j and r_0_ is a correlation threshold that is set to eliminate the weak correlation[28,42,43]. Five different correlation thresholds (r_0_ = 0.15, 0.2, 0.25, 0.3 and 0.35) were computed in this study[35]. The voxel-wise DC value for each individual was converted into a z-score in order to conform to the Gaussian distribution. The z-score maps were smoothed using a 6 mm full-width-at-half-maximum Gaussian kernel.

### Statistical analysis

The demographic and clinical data differences between the OSA patients and GSs were computed using independent two sample *t*-tests with the IBM Statistical Package for the Social Sciences 19.0 software (IBM SPSS Inc., Chicago, IL, USA). We set the significance level at *P* < 0.05.

For the voxel-wise DC, firstly, one sample *t*-tests was performed in each group to identify the spatial centrality distribution (hubs) of the whole brain functional network, respectively. Secondly, we performed independent two-sample *t*-tests using age, ESS, years of educational, and mean FD as nuisance covariates within the default gray matter mask to assess between-group differences in the voxel-wise DC using the REST V1.8. The voxel level of *P* < 0.001, cluster > 40 voxels, AlphaSim corrected, was considered statistically significant.

A linear correlation analysis was performed to evaluate the relationship between clinical variables and the DC value that with significant group differences, controlling for age, and years of education, and mean FD in OSA patients. All statistical analyses were performed using IBM SPSS with a statistical significance level of *P* < 0.05, and the analyses were corrected for multiple comparisons using the Bonferroni correction.

## Results

### Demographic and clinical data

As shown in Table 1, there were no significant differences between the OSA patients and GSs in age, years of education, and mean FD. Significant differences were observed in body mass index, sleep onset latency, sleep efficiency, AHI, SaO_2_ < 90%, rapid eye movement sleep, nadir SaO_2_, AI, ESS, and MoCA between the OSA patients and GSs.

### Degree centrality difference between patients with OSA and GSs

**Fig 1:**
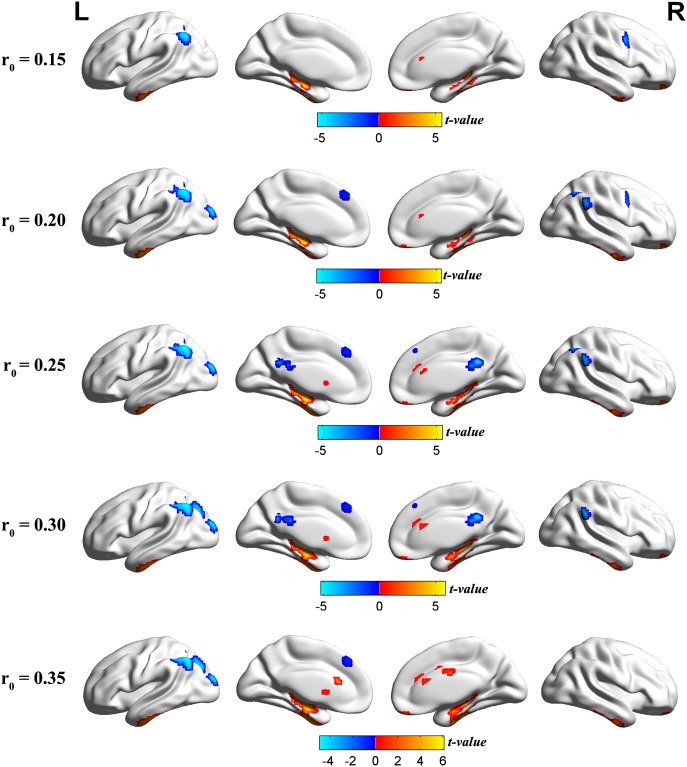
Compared to GSs, the patients with OSA showed remarkably similar altered DC brain areas according to different correlation thresholds (r_0_ = 0.15, 0.2, 0.25, 0.3 and 0.35) (*P* < 0.001, cluster > 40 voxels, AlphaSim corrected). The hot (cool) color indicates significantly increased (decreased) DC brain area. **Abbreviations:** GSs, good sleepers; OSA, obstructive sleep apnea; DC, degree centrality; L(R), left (right) hemisphere.

**Fig 2:**
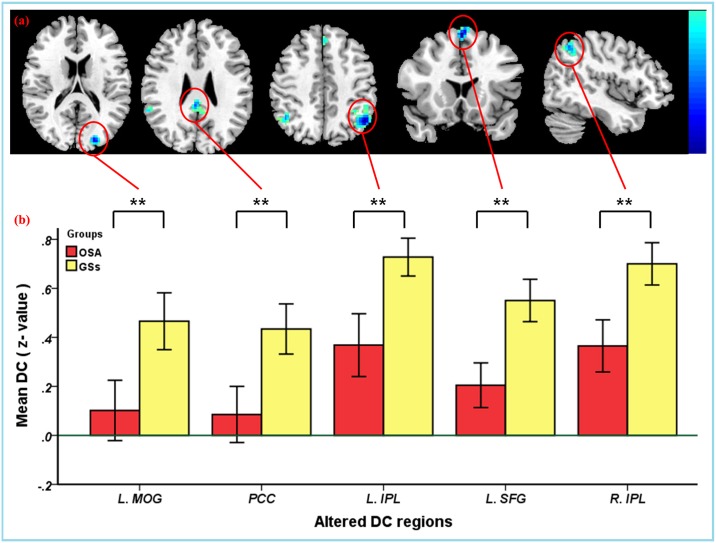
(a) Compared to GSs, the patients with OSA showed reduced DC brain areas when r_0_ was 0.25 (*P* < 0.001, cluster > 40 voxels, AlphaSim corrected). (b) Bar plot of DC for the significant decreased clusters between the patients with OSA and GSs. **Abbreviations:** OSA, obstructive sleep apnea; GSs, good sleepers; DC, degree centrality; MOG, middle occipital gyrus; PCC, posterior cingulate cortex; IPL, inferior parietal lobule; SFG, superior frontal gyrus; L(R), left (right) hemisphere.

**Fig 3:**
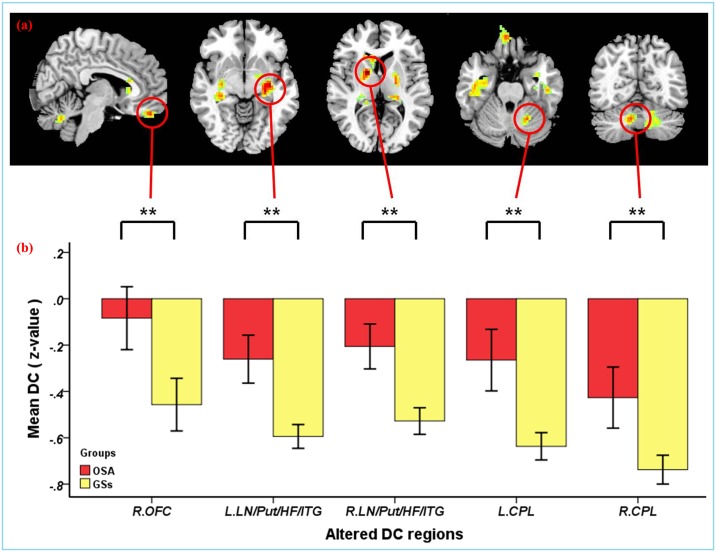
(a) Compared to GSs, the patients with OSA showed increased DC brain areas when r_0_ was 0.25 (*P* < 0.001, cluster > 40 voxels, AlphaSim corrected). (b) Bar plot of DC for the significant increased clusters between the patients with OSA and GSs. **Abbreviations:** OSA, obstructive sleep apnea; GSs, good sleepers; DC, degree centrality; OFC, orbital frontal cortex; LN, lentiform nucleus; Put, putamen; HF, hippocampus formation; ITG, inferior temporal gyrus; CPL, cerebellum posterior lobe; L(R), left (right) hemisphere.

Using spatial distribution maps, highly similar spatial distributions of the functional hubs (high DC) were identified in the two groups, which localized in the frontal lobe, precuneus, parietal lobule, posterior cingulate, occipital lobe, temporal lobe, and cerebellum (S1 Fig), and intergroup differences (Fig 1, S1–S4 Tables) were also remarkably similar based on the different correlation thresholds (r_0_ = 0.15, 0.2, 0.25, 0.3 and 0.35). Therefore, we mainly reported the results for DC when the correlation threshold was 0.25 in a weighted graph. Compared to GSs, the patients with OSA exhibited a significantly decreased DC in the left middle occipital gyrus (MOG), bilateral posterior cingulate cortex (PCC), left superior frontal gyrus (SFG), and bilateral inferior parietal lobule (IPL). However, OSA patients exhibited a significantly increased DC in five clusters. One cluster was located in the right orbital frontal cortex (OFC), two clusters were located in bilateral cerebellum posterior lobes (CPL), the other two clusters were located in bilateral lentiform nucleus (LN) including the putamen, extending to the hippocampus formation (HF), and inferior temporal gyrus (ITG) (Table 2, Figs 2 and 3). These changes of the DC overlapped with the functional hubs. We further examined seed-based FC associated with 5 ROIs (left MOG, PCC, left IPL, left SFG, right IPL) (details in S1 Supplemental Materials). The results were shown in S5 Table and S2 Fig.

**Table 2:** Significant differences in DC between the patients with OSA and GSs (r_0_ = 0.25).

Condition	L/R	Brain regions	MNI coordinates			Cluster size	*t*-value
			X	Y	Z		
OSA<GSs	L	Middle Occipital Gyrus	-21	-87	18	46	-4.91
OSA<GSs	L/R	Posterior Cingulate Cortex	3	-29	27	54	-4.66
OSA<GSs	L	Inferior Parietal Lobule	-48	-60	45	195	-5.40
OSA<GSs	L	Superior Frontal Gyrus	0	18	63	56	-5.43
OSA<GSs	R	Inferior Parietal Lobule	45	-57	48	87	-4.52
OSA>GSs	R	Orbital Frontal Cortex	6	48	-27	66	4.82
OSA>GSs	L	Lentiform Nucleus, Putamen, Hippocampus, Inferior Temporal Gyrus	-33	-15	-6	320	5.21
OSA>GSs	R	Lentiform Nucleus, Putamen, Hippocampus, Inferior Temporal Gyrus	18	3	6	233	5.54
OSA>GSs	L	Cerebellum Posterior Lobe	-19	-56	-25	142	5.21
OSA>GSs	R	Cerebellum Posterior Lobe	9	-63	-33	53	4.63

**Note:** *t-*value, statistical value of peak voxel; (*P* < 0.001, Cluster > 40 voxels, AlphaSim corrected).

**Abbreviations:** OSA, obstructive sleep apnea; GSs, good sleepers; L(R), left (right) hemisphere.

### Correlation between clinical variables and DC

**Fig 4:**
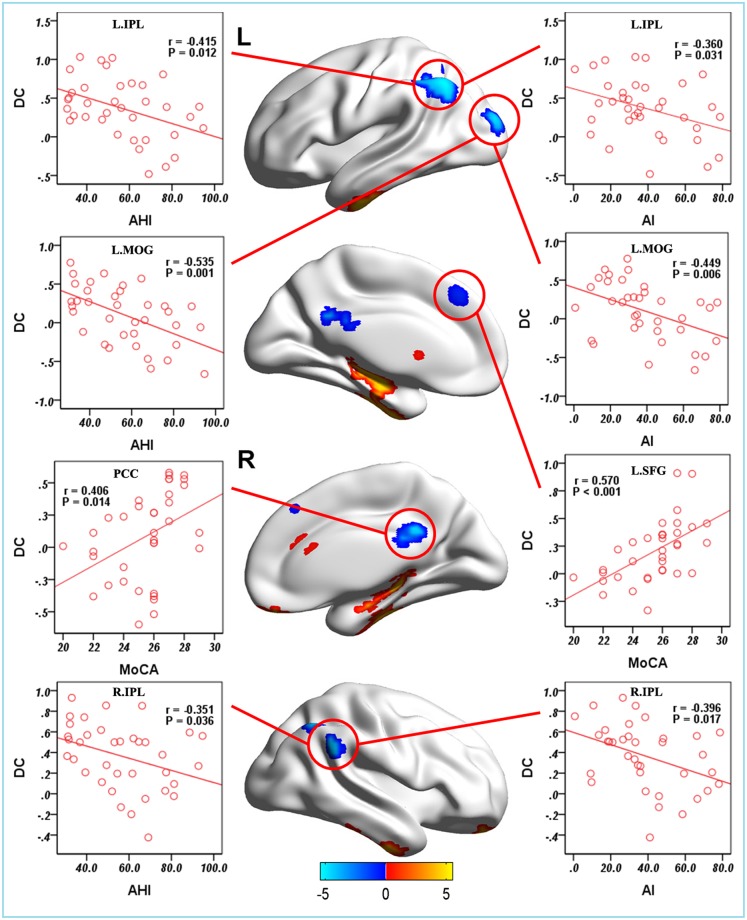
The correlations between the clinical variables and DC in the patients with OSA. **Abbreviations:** OSA, obstructive sleep apnea; GSs, good sleepers; DC, degree centrality; AHI, apnea-hypopnea index; AI, arousal index; IPL, inferior parietal lobule; MOG, middle occipital gyrus; SFG, superior frontal gyrus; PCC, posterior cingulate cortex; L(R), left (right) hemisphere.

In the patients with OSA, a linear correlation analysis showed that AHI was negatively correlated with the DC value in the left MOG (r = -0.535, *P* = 0.001), left IPL (r = -0.415, *P* = 0.012), and right IPL (r = -0.315, *P* = 0.036). AI was negatively correlated with the DC value in the left MOG (r = -0.449, *P* = 0.006), left IPL (r = -0.360, *P* = 0.031), and right IPL (r = -0.396, *P* = 0.017). MoCA was positively correlated with the DC value in the PCC (r = 0.406, *P* = 0.014) and left SFG (r = 0.570, *P* < 0.001) (Fig 4). However, no significant correlation was found between the increased DC and the clinical variables.

## Discussion

We identified the spatial centrality distribution (hubs) of the whole brain functional network and investigated the intrinsic functional hubs changes across the whole brain using voxel-wise DC in male patients with severe OSA. The primary study found remarkably similar spatial distributions of the functional hubs in two groups, which was consistent with previous hub mapping studies [24,28,31,44–46]. However, DC was significantly decreased in the left MOG, left SFG, bilateral IPL, and bilateral PCC, which shared significant overlaps with the DMN, suggesting that these areas are easily damaged, and DC was increased in the right OFC, bilateral CPL, bilateral LN, extending to the HF, and the ITG, which may be an adaptive response related to the other brain damages in the patients with OSA. Furthermore, we also have found that the DC value in bilateral IPL, and left MOG were negatively correlated with AHI and AI, and the DC value in the left SFG and PCC were positively correlated with MoCA in OSA patients. These findings suggest that OSA patients had impaired functional hubs indicating the descending neural network communication and information input, which are related to cognitive dysfunction.

Being male is a major risk factor for OSA, while female OSA patients showed less severe hypoxia, a lower AHI, and more frequently reported anxiety and depression[47]. Previous studies also had demonstrated that anxiety, depression and sex differences might affect the resting-state brain activity[48,49]. In order to avoid the potential confounders of gender differences, anxiety, and depression, only adult male OSA patients were recruited into our study. Excessive daytime sleepiness is a common characteristic of OSA, which might affect the resting-state FC[50]. We compare the group differences in the voxel-wise DC using ESS as a nuisance covariate to reduce the possibility that our rs-FC findings were partially caused by excessive sleepiness. The most important aspect is that previous studies had confirmed that the resting-state FC changes in OSA patients are specific to OSA itself rather than excessive sleepiness[19,21].

In the present study, the DC represents the overall connectivity between particular brain voxels to other brain voxels, which is relatively high in the hubs of the brain network, and plays an important role in information integration, superior information propagation, and critical way stations for information processing, leading to effective information flow[44,46]. In this brain network study, we compared the DC between the two groups and found decreased DC in the bilateral IPL and PCC, which are the core hubs of the DMN that are associated with the extraction of episodic memory[51] collection and evaluation of information[52], mind wandering or daydreaming[53], and attention processing[54]. The finding of DMN abnormalities was not surprising because previous studies had reported this. One previous study showed that OSA patients showed decreased cerebral activation in the left IPL and left PCC during a sustained attention task[55]. OSA patients experience repetitive airway obstruction accompanied by arousal from sleep, resulting in sleep fragmentation. Previous studies showed that male OSA patients had significantly decreased cortical thickness in the left IPL, which correlated with higher respiratory arousals, suggesting that sleep fragmentation is strongly associated with brain tissue damage in the IPL[56]. In this study, we found OSA patients showed higher AHI and AI than GSs. Interestingly, the DC value in the IPL was negatively correlated with AHI and AI, suggesting a strong link between network changes in the IPL and severity and arousal, which indicated that fragmented sleep may be the cause of impaired connectivity of the IPL. As for the PCC, this region showed lower fractional anisotropy[16], lower amplitude of low frequency fluctuation value[18], and dysfunctional connection[20] in patients with OSA. In our study, a positive correlation was observed between DC value in the PCC and MoCA, suggesting that disrupted connectivity in the PCC may underlie the impaired responses in cognitive function during the resting-state.

Consistent with previous structure and metabolism studies[14,57], our current study found significantly reduced DC in the left SFG, located within the prefrontal cortex, which is associated with executive functions[58]. Horne has maintained that the prefrontal cortex is the hardest working region of the brain during wakefulness, necessitating the greatest recovery during sleep[59], and which is differentially sensitive to sleep deprivation and recovery sleep[60]. Disturbances in sleep should result in both diminished performance on measures sensitive to prefrontal cortex functioning and alterations in cerebral response. Indeed, OSA patients also showed damaged prefrontal cortex function with resulting impairment of various executive functions, such as self-regulation of affect and arousal[61]. In this study, we found that reduced DC in the left SFG was positively correlated with MoCA, which indicated that the functional disconnection in the prefrontal cortex play a crucial role in executive dysfunction in OSA patients. The occipital lobe is mainly involved in visual signal processing, but has some influence on memory and motion perception. Recent studies have shown that frontal lobes may participate in processing conceptual memory, while occipital lobes may be involved in perceptual implicit memory in patients with various brain injuries[62]. Our study showed significantly reduced DC in the left MOG and had a negatively correlation with the AHI and AI value in OSA patients, which may be the consequence of repeated episodes of hypoxic exposure and hemodynamic changes.

In addition to the decreased DC in multiple brain regions, we also found significantly increased DC in the bilateral LN including the putamen, extending to the HF and ITG. The LN is an important structure of the basal ganglion and extrapyramidal system, mainly accepting the cerebral cortex and thalamus afferent impulses, and has a wide range of fibers linked with the red nucleus and reticulate structures, which are involved in maintaining muscle tone and muscle activity coordination[63]. These findings may be related to the loss of coordination of the breathing musculature in OSA patients, and may lead to an adaptive increase of upper airway muscle reflective tension to keep the airway unobstructed while awake. Our previous study also found higher regional homogeneity values in the lentiform nucleus including the putamen[23]. The putamen contains dopaminergic neurons, which are involved in mediating emotion and autonomic function[64]. Studies have demonstrated that OSA showed significantly increased global putamen volumes, with regional increased and decreased volume variation, suggesting that the changes result from early and long-standing tissue alterations, which may be due to intermittent hypoxia and impaired perfusion[65]. Our study found that disrupted connectivity in the putamen may result from putamen injury, which has the potential to interfere with normal putamen functioning and to disrupt normal autonomic regulation of the upper airway. Recently, Rosenzweig and his colleagues reported hypertrophy of hippocampus volume and an abnormal connectivity between the hippocampus and the cerebellum in OSA patients[66], which was considered to be an end effect of the neuroglial ischemic preconditioning[67]. In our study, the increased DC in the bilateral CPL and HF may have increased the number of internal connections, and may lead to alterations in a distributed memory system for associative learning for theta oscillations in OSA patients[68]. In addition, our study found increased DC in the right OFC, which was similar to two previous studies, and was identified as important for language[69,70].

### Limitations

There are several limiting factors that should be considered. Firstly, although none of the patients with OSA had a depression or anxiety disorder, the literature reported that OSA was associated with depression and anxiety[71], which may affect the brain’s intrinsic function, but we lack an emotional evaluation. Secondly, this research was a cross-sectional study. A longitudinal study to explore the change in OSA patients before and after treatment may provide more clinical benefit. Finally, DC is the number of direct connections for a node and edges, and represents the local quantifiable measure of a metrics index without an eigenvector. Future study can further explore the qualitative change of global information using voxel-wise eigenvector centrality[28].

## Conclusions

In summary, this is a novel study using voxel-wise DC to investigate the intrinsic functional hubs changes in OSA patients. We found that OSA patients exhibited specific abnormal intrinsic functional hubs, which include relatively reduced DC (e.g., PCC, IPL, left SFG) in response to the destructive aspects and relatively increased DC (e.g., LN, putamen, CPL) that acts as a functional adaptive response. These findings expand our understanding of the functional characteristics of OSA, and may provide new insights into understanding the dysfunction and pathophysiology of OSA patients.

## Supporting Information

S1 Dataset Altered degree centrality between the patients with OSA and good sleepers (r_0_ = 0.25) (including demographic and clinical data). (ZIP)

S1 Fig Highly similar spatial distributions of the functional hubs (high degree centrality) were identified in the two groups, which were localized in the frontal lobe, precuneus, parietal lobule, posterior cingulate, occipital lobe, and temporal lobe, according to different correlation thresholds (r_0_ = 0.15, 0.2, 0.25, 0.3 and 0.35). (TIF)

S2 Fig Significantly decreased regions of seed-based FC in the patients with OSA compared to the GSs (P < 0.001, cluster > 40 voxels, AlphaSim corrected). (TIF)

S1 Supplemental Materials Seed-based functional connectivity analysis. (DOC)

S1 Table Significant differences in DC between the patients with OSA and GSs (r_0_ = 0.15). (DOC)

S2 Table Significant differences in DC between the patients with OSA and GSs (r_0_ = 0.20). (DOC)

S3 Table Significant differences in DC between the patients with OSA and GSs (r_0_ = 0.30). (DOC)

S4 Table Significant differences in DC between the patients with OSA and GSs (r_0_ = 0.35). (DOC)

S5 Table Decreased regions of seed-based FC in OSA patients compared to GSs. (DOC)
