# Supplementary figures and images for: Abnormal Intrinsic Functional Hubs in Severe Male Obstructive Sleep Apnea: Evidence from a Voxel-Wise Degree Centrality Analysis
Source: PLoS One. 2016 Oct 10;11(10):e0164031. doi: 10.1371/journal.pone.0164031 (PMC5056709; doi:10.1371/journal.pone.0164031)

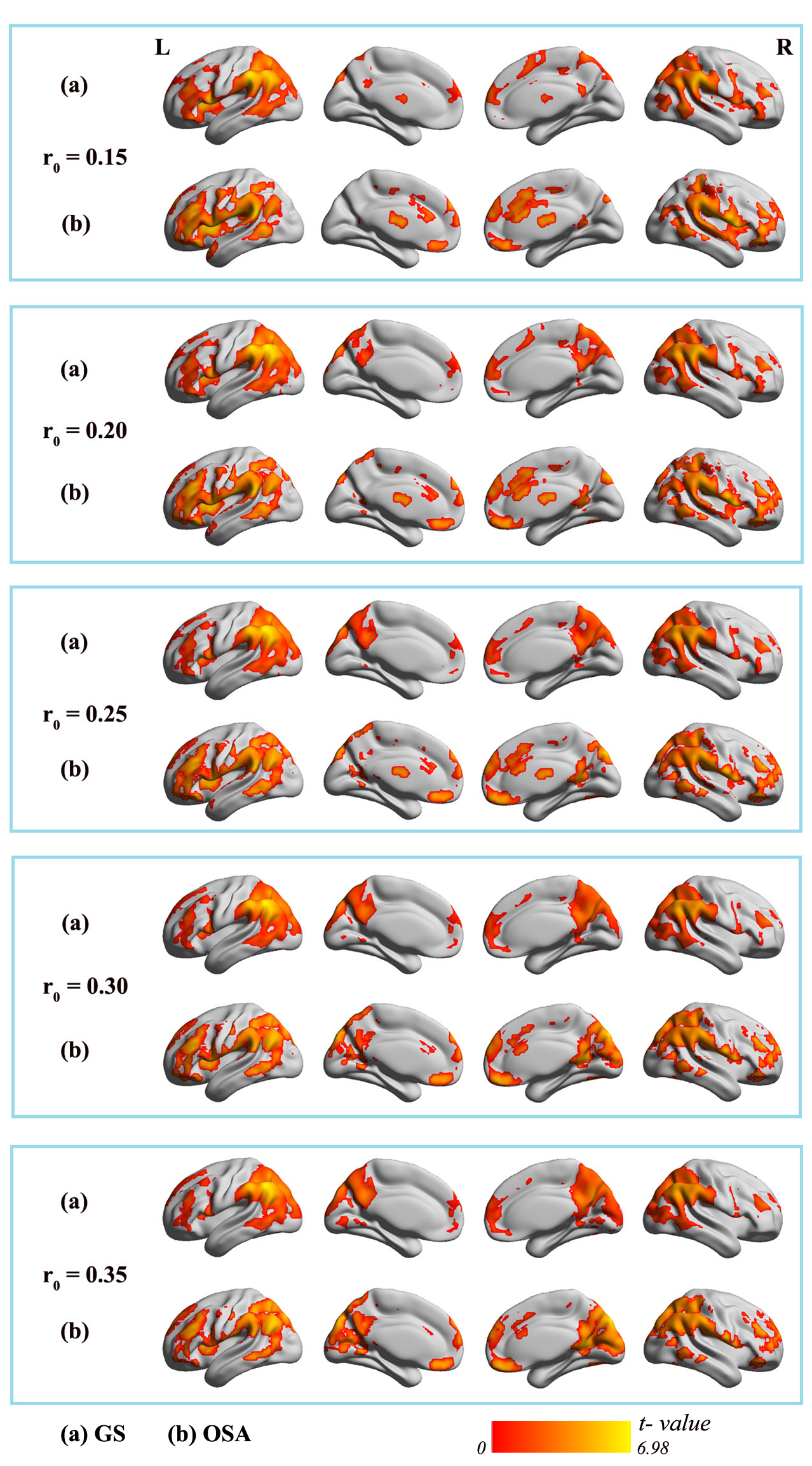

Supplement: S1 Fig — (TIF) [file pone.0164031.s002.tif]

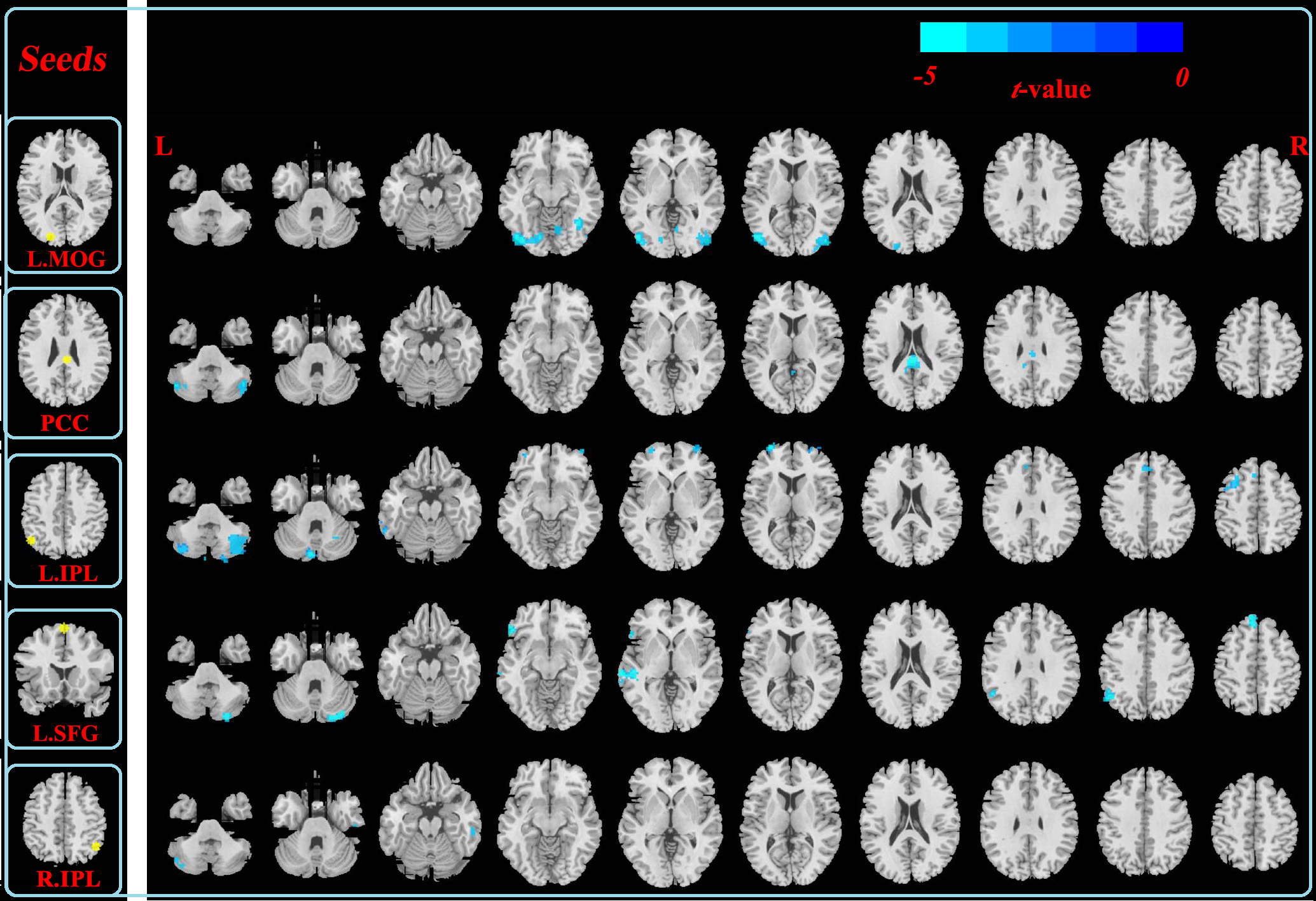

Supplement: S2 Fig — (TIF) [file pone.0164031.s003.tif]
