# Supplementary material for: Abnormal Intrinsic Functional Hubs in Severe Male Obstructive Sleep Apnea: Evidence from a Voxel-Wise Degree Centrality Analysis
Source: PLoS One. 2016 Oct 10;11(10):e0164031. doi: 10.1371/journal.pone.0164031 (PMC5056709; doi:10.1371/journal.pone.0164031)
Supplement: S1 Supplemental Materials — (DOC) [file pone.0164031.s004.doc]

**Seed-based functional connectivity analysis**

1. **Methods in seed-based functional connectivity analysis**

Although the DC analysis can identify voxels that showed altered functional connectivity with other voxels, it cannot provide detailed information regarding the connectivity between a voxel and the particular regions that were changed. Therefore, we used seed-based functional connectivity (FC) approach to further conduct the connectivity abnormality by placing the region-of-interest (ROI) in clusters that exhibited significant reduced DC in patients with OSA. The ROI were chosen in the following: left MOG(x= -21, y= -87 and z= 18), PCC(x= 3, y= -29 and z= 27), left IPL(x= -48, y= -60 and z= 45), left SFG(x= 0, y= 18 and z= 63), right IPL(x= 45 , y= -57 and z= 48). The mean time series of each ROI was extracted, and *Pearson’s* correlation analyses for each individuals were conducted between the seed ROI and the remaining voxels within the gray matter mask. The resulting correlation maps were z-transformed for the group level voxel-wise *t*-test analyses.

1. **Statistical analysis in seed-based functional connectivity**

We performed independent two-sample *t*-tests using age, ESS, years of educational and mean FD as nuisance covariates within the default gray matter mask to assess between-group differences in the seed-based FC using the REST V1.8. The voxel level of P < 0.001, cluster > 40 voxels, AlphaSim corrected, was considered statistically significant.

1. **Results in** **seed-based functional connectivity**

We examined seed-based FC associated with 5 ROIs ( left MOG, PCC, left IPL, left SFG, right IPL) comprising the regions that exhibited reduced DC in the patients with OSA compared to GSs. Seed-based FC analysis found OSA patients demonstrated significantly decreased negative FCs between the those seeds with several brain areas. These nodes included the bilateral occipital lobe, right lingual gyrus, left cuneus, bilateral cerebellum posterior lobe, the PCC, bilateral middle frontal gyrus, left inferior frontal gyrus, left middle temporal gyrus, right inferior temporal gyrus compared to GSs (S5 Table, S2 Fig).

| S5 Table Decreased regions of seed-based FC in OSA patients compared to GSs | | | | | | |
| --- | --- | --- | --- | --- | --- | --- |
| Seeds | Regions | Peak MNI coordinates | | | Number of Voxels | *t*-value |
|  |  | X | Y | Z |  |  |
| L.MOG | L.Occipital Lobe | -42 | -81 | -6 | 310 | -5.035 |
|  | R.Occipital Lobe | 48 | -84 | 3 | 227 | -5.050 |
|  | R.Lingual Gyrus | 6 | -66 | -6 | 50 | -4.527 |
|  | L.Cuneus | -24 | -81 | 15 | 72 | -4.093 |
| PCC | L.Cerebellum Posterior Lobe | -48 | -66 | -39 | 41 | -4.160 |
|  | R.Cerebellum Posterior Lobe | 45 | -63 | -39 | 54 | -4.399 |
|  | Posterior Cingulate | 3 | -33 | 21 | 133 | -4.987 |
| L.IPL | L.Cerebellum Posterior Lobe | -33 | -75 | -45 | 93 | -4.732 |
|  | R.Cerebellum Posterior Lobe | 36 | -72 | -45 | 382 | -5.367 |
|  | L.Middle Temporal Gyrus | -60 | -51 | -18 | 55 | -4.187 |
|  | L.Superior/Middle Frontal Gyrus | -33 | 15 | 60 | 140 | -4.941 |
|  | R.Middle Frontal Gyrus | 45 | 60 | -9 | 58 | -5.025 |
| L.SFG | R.Cerebellum Posterior Lobe | 24 | -84 | -36 | 108 | -4.251 |
|  | L.Inferior Frontal Gyrus | -54 | 33 | -12 | 55 | -4.217 |
|  | L.Middle Temporal Gyrus | -48 | -27 | 0 | 103 | -4.407 |
|  | L.Inferior Parietal Lobule | -51 | -54 | 33 | 81 | -4.040 |
|  | L.Superior Frontal Gyrus | 0 | 18 | 63 | 110 | -4.027 |
| R.IPL | L.Cerebellum Posterior Lobe | -36 | -78 | -42 | 58 | -4.352 |
|  | R.Inferior Temporal Gyrus | 57 | -30 | -24 | 41 | -4.707 |

**Note:** *t-*value, statistical value of peak voxel; (*P* < 0.001, Cluster > 40 voxels, AlphaSim corrected).

**Abbreviations:** OSA, obstructive sleep apnea; GSs, good sleepers; DC, degree centrality; MOG, middle occipital gyrus; PCC, posterior cingulate cortex; IPL, inferior parietal lobule; SFG, superior frontal gyrus; L(R), left (right) hemisphere.


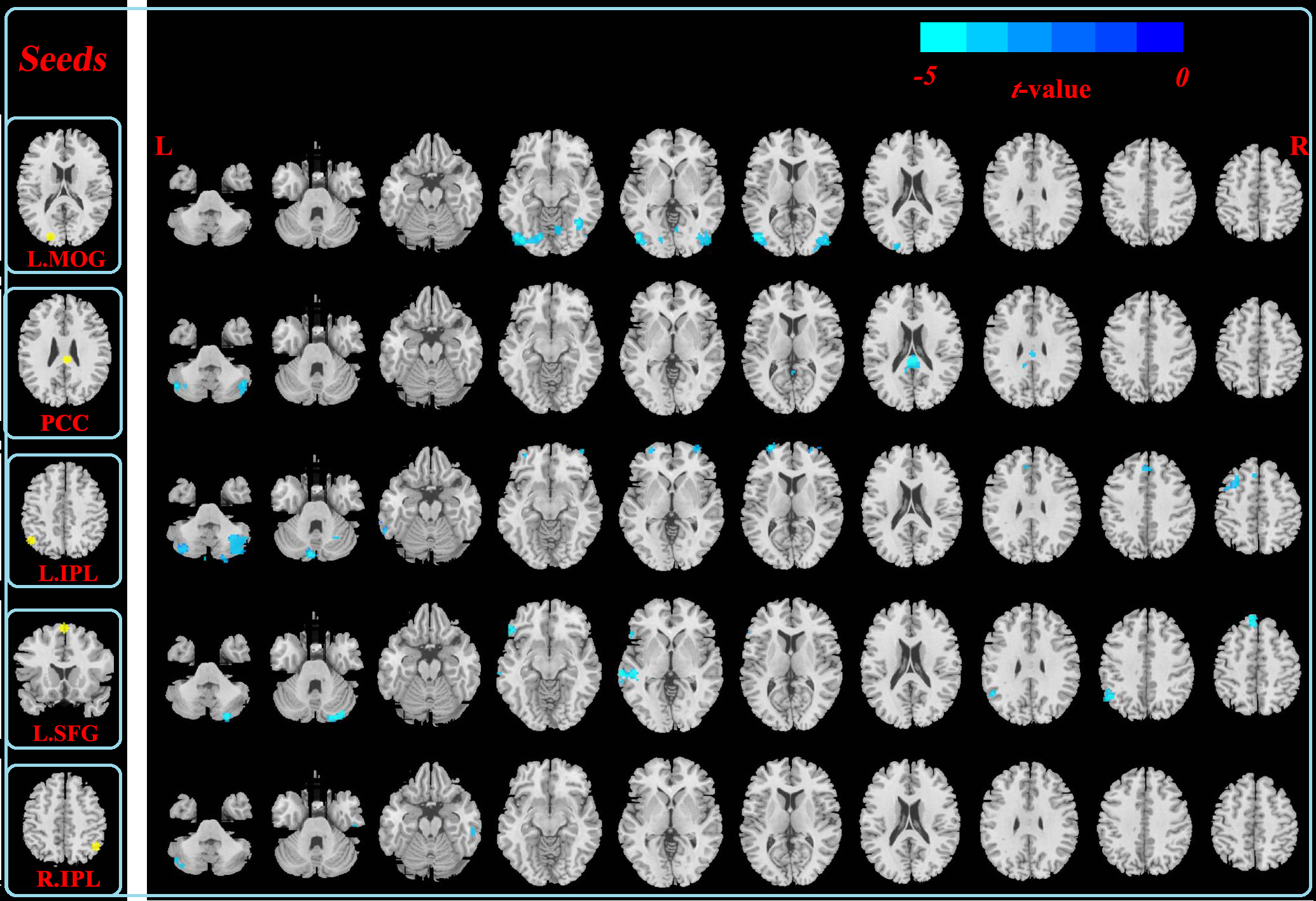
**S2 Fig** Significantly decreased regions of seed-based FC in the patients with OSA compared to the GSs ( *P* < 0.001, cluster > 40 voxels, AlphaSim corrected).

**Abbreviations:** OSA, obstructive sleep apnea; GSs, good sleepers; FC, functional connectivity; MOG, middle occipital gyrus; PCC, posterior cingulate cortex; IPL, inferior parietal lobule; SFG, superior frontal gyrus; L(R), left (right) hemisphere.
