# Supplementary material for: Abnormal Intrinsic Functional Hubs in Severe Male Obstructive Sleep Apnea: Evidence from a Voxel-Wise Degree Centrality Analysis
Source: PLoS One. 2016 Oct 10;11(10):e0164031. doi: 10.1371/journal.pone.0164031 (PMC5056709; doi:10.1371/journal.pone.0164031)
Supplement: S1 Table — (DOC) [file pone.0164031.s005.doc]

| **S1 Table** Significant differences in DC between the patients with OSA and GSs ( r_0_=0.15） | | | | | | | |
| --- | --- | --- | --- | --- | --- | --- | --- |
| Condition | L/R | Brain regions | MNI coordinates | | | Cluster size（Voxle） | *t*-value |
|  |  |  | X | Y | Z |  |  |
| OSA＜GSs | L | Inferior Parietal Lobule | -51 | -57 | 45 | 115 | -5.30 |
| OSA＜GSs | L | Superior Frontal Gyrus | 0 | 18 | 63 | 42 | -5.40 |
| OSA＜GSs | R | Inferior Parietal Lobule | 48 | 6 | 36 | 53 | -4.77 |
| OSA＞GSs | R | Orbital Frontal Cortex | 6 | 48 | -27 | 45 | 4.47 |
| OSA＞GSs | L | Lentiform Nucleus, Putamen, Hippocampus, Inferior Temporal Gyrus | -27 | -24 | -6 | 218 | 5.55 |
| OSA＞GSs | R | Lentiform Nucleus,  Putamen, Hippocampus, Inferior Temporal Gyrus | 15 | 3 | 3 | 209 | 4.76 |
| OSA＞GSs | L | Cerebellum Posterior Lobe | -27 | -57 | -42 | 75 | 4.50 |
