# Supplementary material for: Abnormal Intrinsic Functional Hubs in Severe Male Obstructive Sleep Apnea: Evidence from a Voxel-Wise Degree Centrality Analysis
Source: PLoS One. 2016 Oct 10;11(10):e0164031. doi: 10.1371/journal.pone.0164031 (PMC5056709; doi:10.1371/journal.pone.0164031)
Supplement: S2 Table — (DOC) [file pone.0164031.s006.doc]

| **S2 Table** Significant differences in DC between the patients with OSA and GSs (r_0_=0.20） | | | | | | | |
| --- | --- | --- | --- | --- | --- | --- | --- |
| Condition | L/R | Brain regions | MNI coordinates | | | Cluster size（Voxle） | *t*-value |
|  |  |  | X | Y | Z |  |  |
| OSA＜GSs | L | Middle Occipital Gyrus | -24 | -90 | 18 | 43 | -4.87 |
| OSA＜GSs | L | Superior Frontal Gyrus | 0 | 18 | 63 | 47 | -5.51 |
| OSA＜GSs | L | Inferior Parietal Lobule | -48 | -60 | 45 | 192 | -5.39 |
| OSA＜GSs | R | Middle Frontal Gyrus | 45 | 9 | 51 | 45 | -4.60 |
| OSA＜GSs | R | Inferior Parietal Lobule | 45 | -57 | 48 | 85 | -4.48 |
| OSA＞GSs | R | Orbital Frontal Cortex | 6 | 48 | -27 | 56 | 4.71 |
| OSA＞GSs | L | Lentiform Nucleus,  Putamen, Hippocampus, Inferior Temporal Gyrus | -27 | -24 | -6 | 351 | 5.43 |
| OSA＞GSs | R | Lentiform Nucleus,  Putamen, Hippocampus, Inferior Temporal Gyrus | 15 | 3 | 6 | 301 | 5.11 |
| OSA＞GSs | L | Cerebellum Posterior Lobe | -21 | -57 | -27 | 108 | 4.61 |
| OSA＞GSs | R | Cerebellum Posterior Lobe | 9 | -63 | -33 | 41 | 4.55 |
