# Supplementary material for: Abnormal Intrinsic Functional Hubs in Severe Male Obstructive Sleep Apnea: Evidence from a Voxel-Wise Degree Centrality Analysis
Source: PLoS One. 2016 Oct 10;11(10):e0164031. doi: 10.1371/journal.pone.0164031 (PMC5056709; doi:10.1371/journal.pone.0164031)
Supplement: S3 Table — (DOC) [file pone.0164031.s007.doc]

| **S3 Table** Significant differences in DC between the patients with OSA and GSs (r_0_=0.30） | | | | | | | |
| --- | --- | --- | --- | --- | --- | --- | --- |
| Condition | L/R | Brain regions | MNI coordinates | | | Cluster size（Voxle） | *t*-value |
|  |  |  | X | Y | Z |  |  |
| OSA＜GSs | L | Middle Occipital Gyrus | -21 | -87 | 18 | 46 | -4.91 |
| OSA＜GSs | L/R | Posterior Cingulate, Precuneus | 3 | -39 | 27 | 86 | -4.71 |
| OSA＜GSs | L | Inferior Parietal Lobule | -48 | -60 | 45 | 169 | -5.23 |
| OSA＜GSs | L | Superior Frontal Gyrus | 0 | 18 | 63 | 58 | -5.21 |
| OSA＜GSs | R | Inferior Parietal Lobule | 60 | -45 | 30 | 40 | -4.54 |
| OSA＞GSs | R | Orbital Frontal Cortex | 6 | 48 | -27 | 65 | 4.82 |
| OSA＞GSs | L | Lentiform Nucleus, Putamen, Hippocampus, Inferior Temporal Gyrus | -31 | -15 | -5 | 392 | 5.49 |
| OSA＞GSs | R | Lentiform Nucleus, Putamen, Hippocampus, Inferior Temporal Gyrus | 18 | 3 | 6 | 396 | 5.88 |
| OSA＞GSs | L | Cerebellum Posterior Lobe | -22 | -57 | -27 | 188 | 5.25 |
| OSA＞GSs | R | Cerebellum Posterior Lobe | 9 | -63 | -33 | 68 | 4.78 |
