# Supplementary material for: Abnormal Intrinsic Functional Hubs in Severe Male Obstructive Sleep Apnea: Evidence from a Voxel-Wise Degree Centrality Analysis
Source: PLoS One. 2016 Oct 10;11(10):e0164031. doi: 10.1371/journal.pone.0164031 (PMC5056709; doi:10.1371/journal.pone.0164031)
Supplement: S4 Table — (DOC) [file pone.0164031.s008.doc]

| **S4 Table** Significant differences in DC between the patients with OSA and GSs (r_0_=0.35） | | | | | | | |
| --- | --- | --- | --- | --- | --- | --- | --- |
| Condition | L/R | Brain regions | MNI coordinates | | | Cluster size（Voxle） | *t*-value |
|  |  |  | X | Y | Z |  |  |
| OSA＜GSs | L | Middle Occipital Gyrus | -21 | -87 | 18 | 41 | -4.77 |
| OSA＜GSs | L/R | Precuneus | -27 | -72 | 33 | 45 | -4.41 |
| OSA＜GSs | L | Inferior Parietal Lobule | -48 | -60 | 45 | 132 | -4.96 |
| OSA＜GSs | L | Superior Frontal Gyrus | 0 | 18 | 63 | 51 | -4.92 |
| OSA＞GSs | R | Orbital Frontal Cortex | 6 | 48 | -27 | 55 | 4.75 |
| OSA＞GSs | L | Lentiform Nucleus, Putamen, Hippocampus, Inferior Temporal Gyrus | -31 | -12 | -12 | 432 | 5.61 |
| OSA＞GSs | R | Lentiform Nucleus, Putamen, Hippocampus, Inferior Temporal Gyrus | 18 | 3 | 6 | 437 | 6.11 |
| OSA＞GSs | L | Cerebellum Posterior Lobe | -9 | -78 | -45 | 305 | 5.61 |
| OSA＞GSs | R | Cerebellum Posterior Lobe | 18 | -60 | -54 | 69 | 5.00 |
