# Supplementary material for: Abnormal Intrinsic Functional Hubs in Severe Male Obstructive Sleep Apnea: Evidence from a Voxel-Wise Degree Centrality Analysis
Source: PLoS One. 2016 Oct 10;11(10):e0164031. doi: 10.1371/journal.pone.0164031 (PMC5056709; doi:10.1371/journal.pone.0164031)
Supplement: S5 Table — (DOC) [file pone.0164031.s009.doc]

| S5 Table Decreased regions of seed-based FC in OSA patients compared to GSs | | | | | | |
| --- | --- | --- | --- | --- | --- | --- |
| Seeds | Regions | Peak MNI coordinates | | | Number of Voxels | *t*-value |
|  |  | X | Y | Z |  |  |
| L.MOG | L.Occipital Lobe | -42 | -81 | -6 | 310 | -5.035 |
|  | R.Occipital Lobe | 48 | -84 | 3 | 227 | -5.050 |
|  | R.Lingual Gyrus | 6 | -66 | -6 | 50 | -4.527 |
|  | L.Cuneus | -24 | -81 | 15 | 72 | -4.093 |
| PCC | L.Cerebellum Posterior Lobe | -48 | -66 | -39 | 41 | -4.160 |
|  | R.Cerebellum Posterior Lobe | 45 | -63 | -39 | 54 | -4.399 |
|  | Posterior Cingulate | 3 | -33 | 21 | 133 | -4.987 |
| L.IPL | L.Cerebellum Posterior Lobe | -33 | -75 | -45 | 93 | -4.732 |
|  | R.Cerebellum Posterior Lobe | 36 | -72 | -45 | 382 | -5.367 |
|  | L.Middle Temporal Gyrus | -60 | -51 | -18 | 55 | -4.187 |
|  | L.Superior/Middle Frontal Gyrus | -33 | 15 | 60 | 140 | -4.941 |
|  | R.Middle Frontal Gyrus | 45 | 60 | -9 | 58 | -5.025 |
| L.SFG | R.Cerebellum Posterior Lobe | 24 | -84 | -36 | 108 | -4.251 |
|  | L.Inferior Frontal Gyrus | -54 | 33 | -12 | 55 | -4.217 |
|  | L.Middle Temporal Gyrus | -48 | -27 | 0 | 103 | -4.407 |
|  | L.Inferior Parietal Lobule | -51 | -54 | 33 | 81 | -4.040 |
|  | L.Superior Frontal Gyrus | 0 | 18 | 63 | 110 | -4.027 |
| R.IPL | L.Cerebellum Posterior Lobe | -36 | -78 | -42 | 58 | -4.352 |
|  | R.Inferior Temporal Gyrus | 57 | -30 | -24 | 41 | -4.707 |

**Note:** *t-*value, statistical value of peak voxel; (*P* < 0.001, Cluster > 40 voxels, AlphaSim corrected).

**Abbreviations:** OSA, obstructive sleep apnea; GSs, good sleepers; DC, degree centrality; MOG, middle occipital gyrus; PCC, posterior cingulate cortex; IPL, inferior parietal lobule; SFG, superior frontal gyrus; L(R), left (right) hemisphere.
